# Early Prediction of Sepsis From Clinical Data: The PhysioNet/Computing in Cardiology Challenge 2019

**DOI:** 10.1097/CCM.0000000000004145

**Published:** 2020-01-15

**Authors:** Matthew A. Reyna, Christopher S. Josef, Russell Jeter, Supreeth P. Shashikumar, M. Brandon Westover, Shamim Nemati, Gari D. Clifford, Ashish Sharma

**Affiliations:** 1 Department of Biomedical Informatics, Emory University, Atlanta, GA.; 2 Department of Biomedical Engineering, Georgia Institute of Technology, Atlanta, GA.; 3 Department of Biomedical Informatics, University of California San Diego, San Diego, CA.; 4 Department of Neurology, Massachusetts General Hospital, Boston, MA.

**Keywords:** competition, early detection and treatment, evaluation metrics, generalizability, open-source algorithms, PhysioNet, sepsis, sequential prediction tasks

## Abstract

Sepsis is a major public health concern with significant morbidity, mortality, and healthcare expenses. Early detection and antibiotic treatment of sepsis improve outcomes. However, although professional critical care societies have proposed new clinical criteria that aid sepsis recognition, the fundamental need for early detection and treatment remains unmet. In response, researchers have proposed algorithms for early sepsis detection, but directly comparing such methods has not been possible because of different patient cohorts, clinical variables and sepsis criteria, prediction tasks, evaluation metrics, and other differences. To address these issues, the PhysioNet/Computing in Cardiology Challenge 2019 facilitated the development of automated, open-source algorithms for the early detection of sepsis from clinical data.

**Design:**

Participants submitted containerized algorithms to a cloud-based testing environment, where we graded entries for their binary classification performance using a novel clinical utility-based evaluation metric. We designed this scoring function specifically for the Challenge to reward algorithms for early predictions and penalize them for late or missed predictions and for false alarms.

**Setting:**

ICUs in three separate hospital systems. We shared data from two systems publicly and sequestered data from all three systems for scoring.

**Patients:**

We sourced over 60,000 ICU patients with up to 40 clinical variables for each hour of a patient’s ICU stay. We applied Sepsis-3 clinical criteria for sepsis onset.

**Interventions:**

None.

**Measurements and Main Results:**

A total of 104 groups from academia and industry participated, contributing 853 submissions. Furthermore, 90 abstracts based on Challenge entries were accepted for presentation at Computing in Cardiology.

**Conclusions:**

Diverse computational approaches predict the onset of sepsis several hours before clinical recognition, but generalizability to different hospital systems remains a challenge.

## Abstract 2

Supplemental Digital Content is available in the text.

Sepsis is a life-threatening condition that occurs when the body’s response to infection causes tissue damage, organ failure, or death (1–3). In the United States, nearly 1.7 million people develop sepsis and 270,000 people die of sepsis each year; over one third of people who die in U.S. hospitals have sepsis (4). Globally, an estimated 30 million people develop sepsis and 6 million people die of sepsis each year (5). Costs for managing sepsis in U.S. hospitals exceed those for any other health condition at $24 billion annually (13% of U.S. healthcare expenses); a majority of these costs are for patients who develop sepsis during their hospital stay (6). The developing world faces additional expenses from sepsis management and higher risks of adverse outcomes. Altogether, sepsis is a major public health issue responsible for significant morbidity, mortality, and healthcare expenses (7–10).

The reliable and early identification of sepsis is often complicated by its syndromic nature, which can contribute to delays in treatment. The importance of early identification and treatment of sepsis is highlighted in two recent studies that suggest an increase in the adjusted mortality of septic patients who experienced delays in antibiotic therapy (11, 12). This effect is even more profound in patients suffering from septic shock, where hourly delays were associated with an 3.6–9.9% increase in mortality per hour (13). Professional critical care societies have proposed clinical criteria for recognizing and treating sepsis (1–3); however, the fundamental need for early and reliable identification of sepsis remains unmet (14).

The PhysioNet/Computing in Cardiology Challenge is an international competition focused on open-source solutions for complex physiologic signal processing and medical classification problems (15). In 2019, the Challenge’s 20th year, we asked participants to develop automated techniques for the early detection of sepsis from clinical data.

Computational approaches promise to improve the early detection of sepsis. Such approaches typically apply machine learning techniques to clinical data (e.g., see Refs. [16–18]), with the goal of making real-time predictions up to a day before clinical recognition of sepsis. However, the relative strengths and weaknesses of algorithmic approaches are unclear for a variety of reasons. The PhysioNet/Computing in Cardiology Challenge 2019 provided an opportunity to explore the limits of computational approaches for detecting sepsis.

First, algorithms for the early detection of sepsis frequently address subtly different problems, and they tend to have been developed and tested in different patient cohorts with different clinical variables and labels arising from different clinical criteria for sepsis. For the Challenge, we provided a common problem statement using the same clinical variables and sepsis criteria. We shared data from two separate hospital systems and sequestered data from a third hospital system. Algorithms that overfit on the shared databases typically underperformed on the hidden database, particularly if they encoded data collection behaviors specific to a given hospital system. Furthermore, we ran algorithms only once on the full hidden dataset to prevent sequential training on the hidden data, and we compared algorithms to identify teams that attempted to circumvent the rules and have more “bites of cherry” than other teams.

Second, different studies often employ different evaluation metrics, and such metrics do not necessarily reflect the clinical utility of sepsis detection and treatment. Traditional scoring metrics, such as area under the curve (AUC) metrics, do not explicitly reward early detection or penalize false alarms or overtreatment. For the Challenge, we devised a novel evaluation metric that addresses these issues and could be generally applicable to predicting infrequent events in time series data.

Third, the complexity of such algorithms is nearly impossible to adequately describe in a research article. For the Challenge, we encouraged and facilitated the open sourcing of algorithms to ensure that subtle implementation details are provided and reproducible.

## METHODS

### Challenge Objective

The goal of this Challenge was the development of algorithms for the early prediction of sepsis using routinely available clinical data. Early predictions of sepsis are potentially lifesaving, whereas late or missed predictions are potentially life threatening, and false alarms consume hospital resources and erode trust in the algorithms themselves (19).

For this Challenge, we asked participants to design and implement working, open-source algorithms that can, based only on the provided clinical data, automatically identify a patient’s risk of sepsis and make a positive or negative prediction of sepsis for every hourly time window in the patient’s clinical record. In particular, we asked participants to predict sepsis at least 6 hours (but not more than 12 hr) before the onset time of sepsis according to Sepsis-3 clinical criteria (1–3). To evaluate each algorithm, we designed a new clinical utility-based scoring metric that rewards algorithms for early sepsis predictions and penalizes them for late and missed sepsis predictions as well as for false alarms. The winners of this Challenge were the team whose algorithm gave predictions with the highest clinical utility score for patients in a hidden test set across three hospital systems.

We awarded prizes to teams with winning algorithms. Although we allowed both noncommercial and commercial entities to enter, only open-source entries were eligible for prizes. All code was required to be submitted to ensure that methods were replicable and because no teams had access to the hidden data. This allowed for the comparison of winning teams with commercial entities and increased the competitive landscape.

### Challenge Data

We obtained the data for the Challenge from three geographically distinct U.S. hospital systems with three different Electronic Medical Record (EMR) systems: Beth Israel Deaconess Medical Center (hospital system A), Emory University Hospital (hospital system B), and a third, unidentified hospital system (hospital system C). These data were collected over the past decade with approval from the appropriate institutional review boards. We deidentified and labeled the data using Sepsis-3 clinical criteria (1–3). Data and labels for 40,336 patients from hospital systems A and B were posted publicly for download, and data and labels for 24,819 patients from hospital systems A, B, and C were sequestered as hidden test sets.

The Challenge data consisted of a combination of hourly vital sign summaries, laboratory values, and static patient descriptions. In particular, the data contained 40 clinical variables: eight vital sign variables, 26 laboratory variables, and six demographic variables; **Table 1** describes these variables. Altogether, these data included over 2.5 million hourly time windows and 15 million data points.

**TABLE 1.**
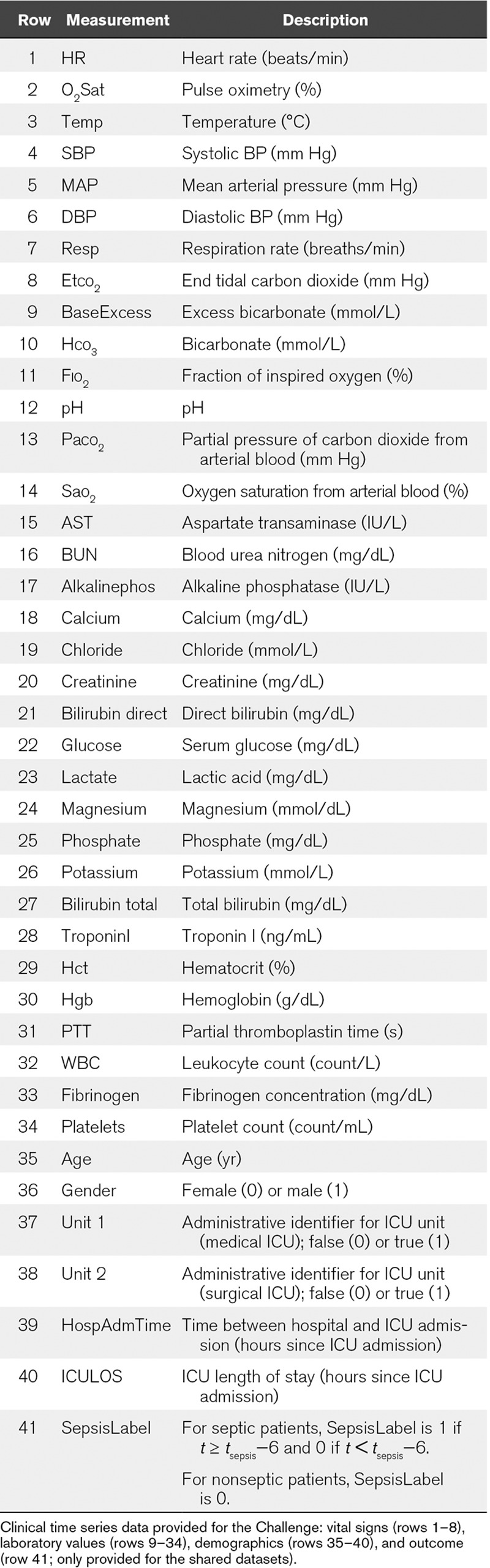
Feature Summary

Data extracted from the EMR underwent a series of preprocessing steps prior to formal analysis and model development. All patient features were condensed into hourly bins simplifying model development and testing; for example, multiple heart rate measurements in an hourly time window were summarized as the median heart rate measurement. Multiple Logical Observation Identifiers Names and Codes codes describing the same clinical parameter were condensed into a single variable; for example, serum hemoglobin and arterial hemoglobin became hemoglobin.

We labeled patient data in accordance with Sepsis-3 clinical criteria (1–3). For each septic patient, we specified the following three time points to define the onset time *t*_sepsis_ of sepsis:

- *t*_suspicion_: Clinical suspicion of infection identified as the earlier timestamp of IV antibiotics and blood cultures within a given time interval. If IV antibiotics were given first, then the cultures must have been obtained within 24 hours. If cultures were obtained first, then IV antibiotic must have been ordered within 72 hours. In either case, IV antibiotics must have been administered for at least 72 consecutive hours.
- *t*_SOFA_: Occurrence of organ failure as identified by a two-point increase in the Sequential Organ Failure Assessment (SOFA) score within a 24-hour period.
- *t*_sepsis_: Onset of sepsis identified as the earlier of *t*_suspicion_ and *t*_SOFA_ as long as *t*_SOFA_ occurred no more than 24 hours before or 12 hours after *t*_suspicion_.

Missing and erroneous data were intentionally preserved as part of the Challenge. However, patients with less than 8 hourly time windows of data in the ICU were not included, and patients with *t*_sepsis_ less than 4 hours after ICU admission were not included. Patient records were truncated after ICU discharge, and patients with more than 2 weeks of hourly time windows were truncated to 2 weeks.

**Figure 1.**
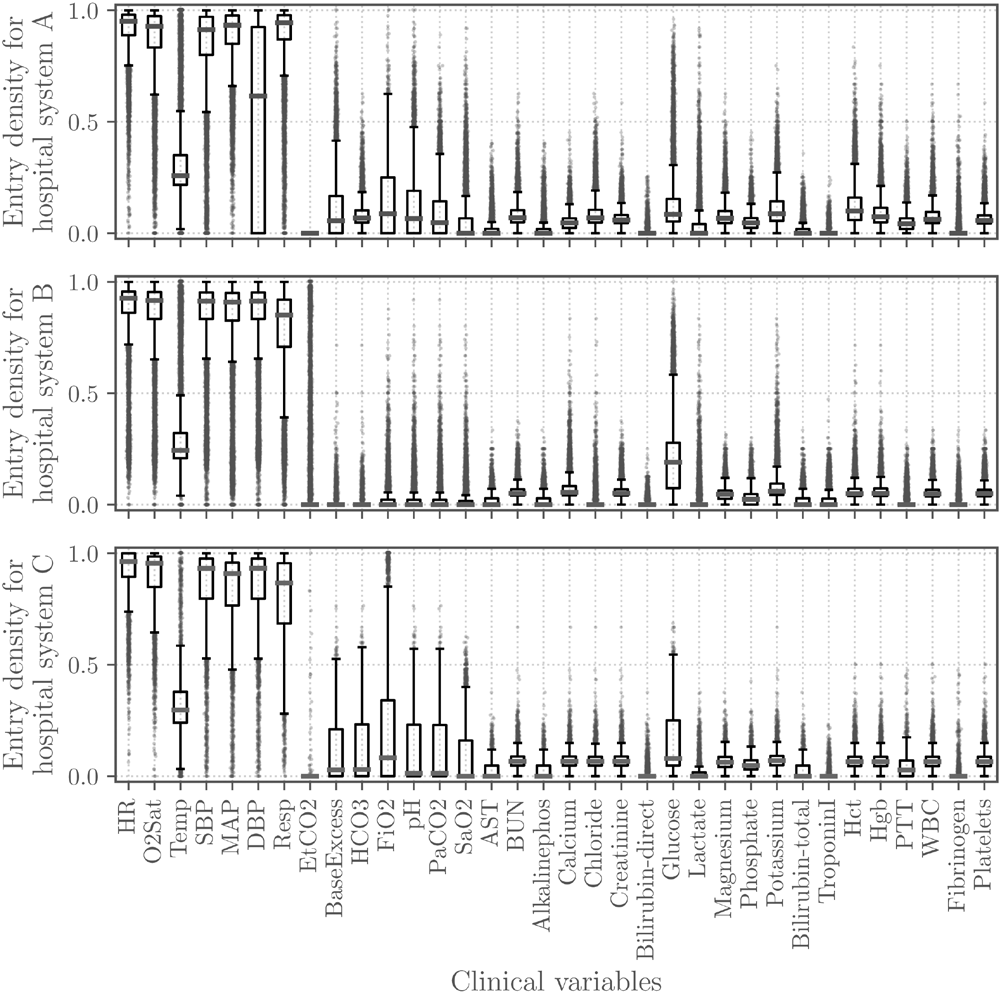
Densities of vital sign (rows 1-8) and laboratory value (rows 9-34) entries (fraction of non-empty entries) in the shared and hidden datasets for hospital systems A, B, and C.

**Supplemental Table 1** (Supplemental Digital Content 1, http://links.lww.com/CCM/F206) summarizes the datasets for the two shared hospital databases. **Figure 1** shows the densities of entries (i.e., the fraction of non-empty hourly measurements) for each vital sign and laboratory value in each patient record; most vital signs were updated on an hourly basis in most patient records, and most laboratory values were updated on a daily basis. **Supplemental Figure 1** (Supplemental Digital Content 2, http://links.lww.com/CCM/F207) shows the distributions of these entries across patient records. **Supplemental Figure 2** (Supplemental Digital Content 3, http://links.lww.com/CCM/F208) quantifies the difference between the vital sign and laboratory value distributions between hospital systems using Jensen-Shannon divergence. Note that most clinical variables have similar distributions across hospital systems.

### Challenge Scoring

We scored each algorithm’s predictions using a novel evaluation metric that we created for the Challenge. To better capture the clinical utility of sepsis detection and treatment, this metric rewarded algorithms for early sepsis predictions in septic patients, and it penalized algorithms for late or missed sepsis predictions in septic patients and for sepsis predictions in nonseptic patients.

Each algorithm made a binary sepsis prediction for each hourly time window in each patient record. To evaluate each algorithm, we first defined a score for each prediction and then aggregated these scores over all hourly time windows and all patient records.

**Figure 2.**
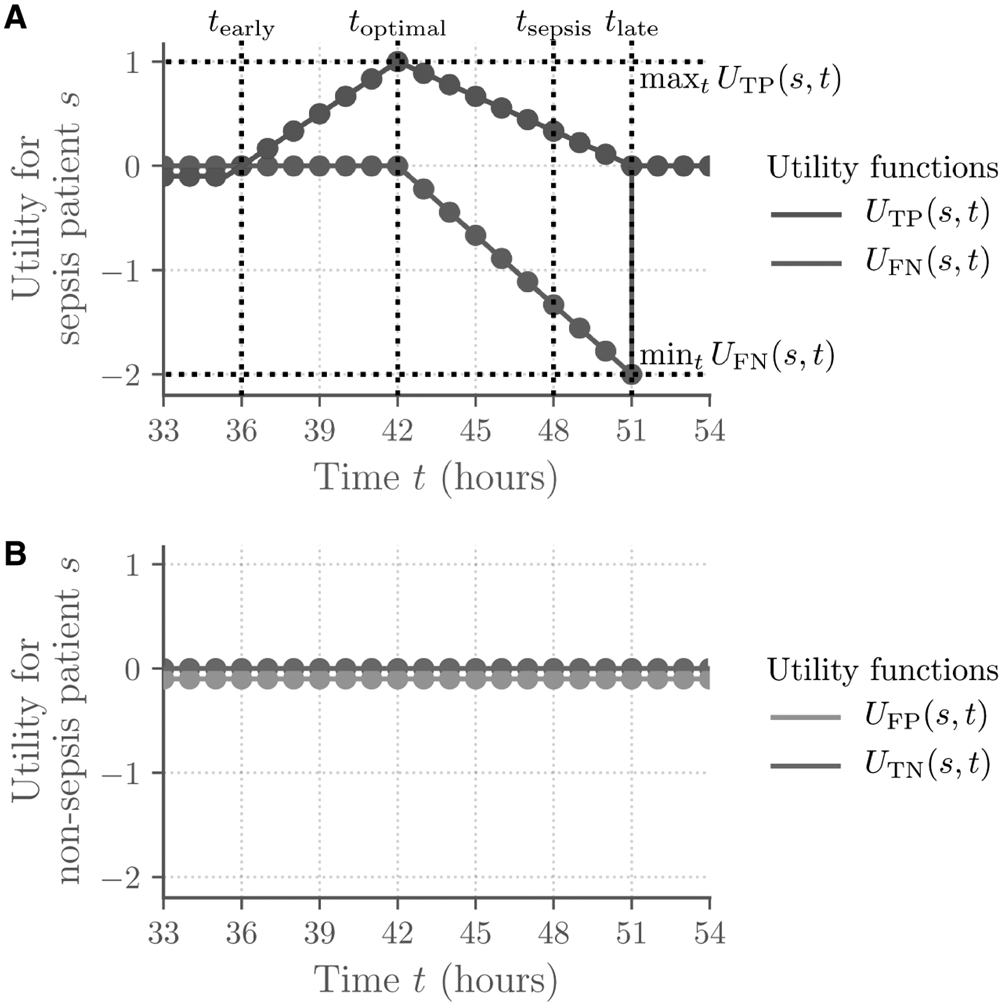
Diagrams of utility of positive and negative predictions for sepsis and non-septic patients; the time *t*_sepsis_ = 48 of sepsis onset is given as an example.

Given an algorithm’s prediction for an hourly time window *t* in a patient record *s*, we define a score


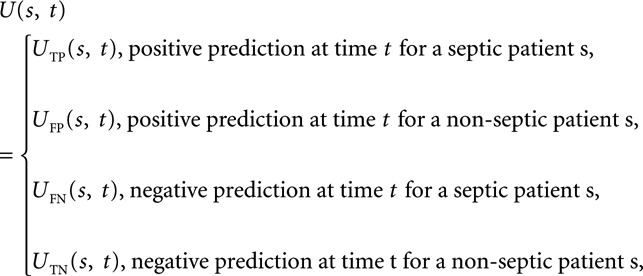
[1]

where *U*_TP_(*s, t*), *U*_FP_(*s, t*), *U*_FN_(*s, t*), and *U*_TN_(*s, t*) are illustrated in **Figure 2*A*** for an example septic patient and in **Figure 2*B*** for an example nonseptic patient. These scores were chosen to reflect the broad clinical realities of sepsis detection and treatment, and the actual utility values and time points in [1] and Figure 2 can be chosen to capture the specific preferences or trade-offs of any particular hospital system.

For patients who become septic during their ICU stay, early sepsis detection tends to be beneficial. Therefore, sepsis predictions in septic patients who were at least 12 hours before and at most 3 hours after the onset time *t*_sepsis_ of sepsis were rewarded with a maximum reward at 6 hours before *t*_sepsis_, and sepsis predictions that are more than 12 hours before *t*_sepsis_ were slightly penalized. Similarly, for patients who become septic during their ICU stay, very early predictions may be implausible or unhelpful, and late or missed septic predictions are generally harmful. Therefore, sepsis predictions in septic patients who were more than 12 hours before *t*_sepsis_ were slightly penalized, and nonsepsis predictions that were less than 6 hours before *t*_sepsis_ were increasingly penalized.

For patients who do not become septic during their ICU stay, sepsis predictions contribute to alarm fatigue and lower confidence in algorithms, antibiotic overuse, and overall poor allocation of hospital attention and resources. Therefore, sepsis predictions in nonseptic patients were slightly penalized. Similarly, nonsepsis predictions in nonseptic patients were neither rewarded nor penalized.

Given an algorithm’s predictions for all hourly time windows *T* (*s*) in each patient record *s*, we define the total score for an algorithm as the sum


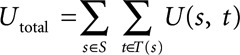
[2]

over all predictions. For easier interpretability, we normalize [2] so that the optimal algorithm with the highest possible score receives a normalized score of 1 and a completely inactive algorithm that only makes nonsepsis predictions receives a normalized score of 0, that is,


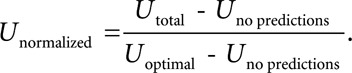
[3]

Each algorithm received a score from [3], and the algorithm with the highest value of [3] on the full sequestered dataset from hospital systems A, B, and C won the Challenge.

### Challenge Submissions

Challenge participants submitted their algorithms for evaluation on the sequestered data. This strategy encouraged reproducibility and gave participants the ability to validate their algorithms on real-world datasets.

Each team was allowed a total of five scored entries during an unofficial phase of the Challenge from February 8, 2019, to April 14, 2019. This phase allowed for beta testing and socialization of the submission system, rules, and scoring mechanism, and teams were required to submit at least one entry during the unofficial phase for Challenge eligibility. Subsequently, each eligible team was allowed a total of 10 scored entries during the official phase of the Challenge from April 25, 2019, to August 25, 2019. This phase allowed teams to submit their models for evaluation on test data from hospital system A; scoring on the full hidden test data occurred only after the official phase at the end of the Challenge. This limit also improved the tractability of the Challenge. Because we did not heavily restrict the languages and libraries that teams could use, many teams required technical support for their submissions.

The submission system relied on containers that were orchestrated, as pipelines, on the Google Cloud Platform; **Supplemental Figure 3** (Supplemental Digital Content 4, http://links.lww.com/CCM/F209) illustrates this system. A container is a standard unit of software that packages code and its dependencies so that the application runs readily and reliably in different computing environments. For the Challenge, we used the Docker containerization environment. Participants packaged their entries and uploaded them to a GitHub repository (Microsoft, San Francisco, CA), which was shared privately with the Challenge organizers. For each submission, the submission system cloned the repository, created a pipeline that consisted of the entry and our scoring function, and launched this pipeline on Google Cloud. This system allowed us to score multiple entries in parallel. During the unofficial and official phases of the Challenge, we processed over a thousand submissions in Julia (https://julialang.org), MATLAB (MathWorks, Natick, MA), Python (https://www.python.org), and R (R Foundation for Statistical Computing, Vienna, Austria; https://www.r-project.org) from over a hundred participants.

Each entry was run in a virtual machine with two central processing units and 12 GB of random access memory, and each entry was allowed 24 hours of run time on each hidden test set. The submission system orchestrator, Cromwell (The Eli and Edythe L. Broad Institute of MIT and Harvard, Cambridge, MA), typically requested a n2-highmem-2 machine type on Google Cloud.

### Implementations of Evaluation Metric and Baseline Model

To provide a baseline model, we trained a Weibull-Cox regression model and provided open-source implementations of this model in Julia, MATLAB, Python, and R. These implementations also served as examples of how to devise a working prediction algorithm in each language that we accepted for the Challenge. We also provided open-source implementations of our clinical utility-based scoring function. The code is available online at https://github.com/physionetchallenges.

### Analysis of Entry Independence, Collusion, and Plagiarism

After the conclusion of each Challenge, we frequently build a meta-algorithm from the final entries that are weighted by their independence; agreement between highly similar algorithms can suggest a false consensus of predictions. To increase the independence of algorithms, we therefore prohibited teams from collaboration at any point of the Challenge. Specifically, we note the following:

- Multiple teams from a single entity (such as a company, university, or department) were permitted as long as the teams were truly independent and did not share team members, code, or ideas at any point. Multiple teams from the same research group or unit within a company were not allowed because we did not believe that true independence between teams could be maintained when team members may frequently interact.
- New team members could join as long as they had not previously been involved with another team or had communicated with a team member from another team concerning this year’s Challenge.
- Teams could use public code if it had been posted before the competition. Members of teams were not allowed to publicly post code during the competition or use another competitor’s code that was posted during the competition whether or not it was intentionally made public.
- Members of teams were not allowed to publicly post information describing their methods or give a talk outside of their own research group at any point during the competition that revealed the methods they have employed or planned to employ in the Challenge. Members of teams were allowed to present or publish on methods on other data as long as they did not indicate that they planned to apply it to Challenge data until after the competition.
- Members of teams were required to use the same team name and email address throughout the course of the competition, including for abstract submissions to the public forum at which they defended their work, that is, at Computing in Cardiology.

Although the rules of the Challenge strictly prohibited teams from more than 10 scored entries during the official phase of the Challenge, several entries from apparently different teams achieved exactly the same score. An investigation of their submissions showed strong similarities between these teams, which, when questioned, either did not reply or claimed not to have colluded. By examining associations among email addresses, team names, and GitHub repositories, we were able to identify several prohibited collaborations. **Supplemental Figure 4** (Supplemental Digital Content 5, http://links.lww.com/CCM/F210) illustrates associations among email addresses, team names, and GitHub users from Challenge submissions, where each team was expected to have only one email address, team name, and GitHub user. Some associations with multiple email addresses, team names, and/or GitHub users indicated prohibited collaborations and resulted in disqualifications.

### Results

A total of 104 teams from academia and industry submitted a total of 853 entries during the official phase of the Challenge; of these, 88 distinct teams with a total of 430 entries were able to be scored. Recall that each team received training data and labels for hospital systems A and B but not for hospital system C. Each successful entry received scores on the test data for hospital system A during the unofficial and official phases of the Challenge, and each team nominated its favorite successful entry for evaluation on the full test data containing patient records from hospital systems A, B, and C. **Table 2** summarizes the teams with the highest-scoring entries.

**TABLE 2.**
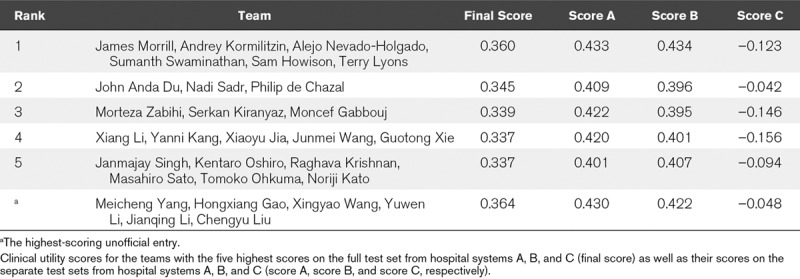
Top Clinical Utility Scores

By curating clinical data from multiple hospital systems and sharing different amounts of data and information from these systems, we demonstrated that algorithms generally performed much better in two hospital systems for which we provided training data than a third hospital system for which we provided no training data.

**Figure 3.**
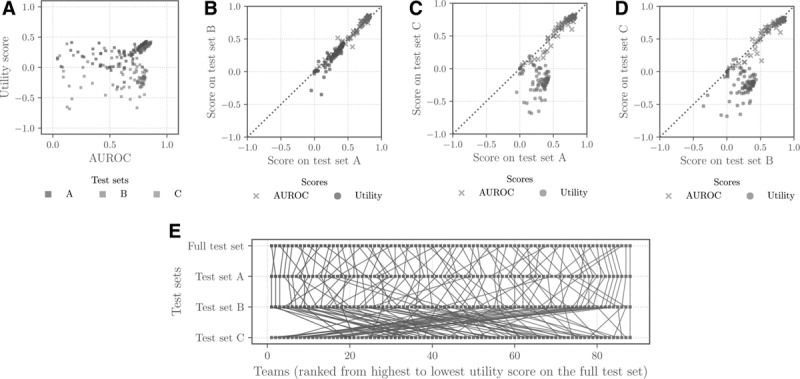
Comparison of each algorithm’s AUROC and utility scores on test data from hospital systems A, B, and C, where we shared training data for hospital systems A and B but not for hospital system C. A, Comparison of each algorithm’s area under the receiver operating characteristic curve (AUROC) and utility scores on test sets A, B, and C. B, Comparison of each algorithm’s AUROC and utility scores on test sets A and B. C, Comparison of each algorithm’s AUROC and utility scores on test sets A and C. D, Comparison of each algorithm’s AUROC and utility scores on test sets B and C. E, Ranked performance of the final algorithms on test sets A, B, and C. Red indicates a high overall ranking across all three databases, and blue indicates a low overall ranking. Lines from top to bottom indicate how the individual algorithm ranking changed when considering the performance on each database. Algorithms that performed well on test sets A and B generally performed relatively poorly on test set C.

Although algorithms that performed well by one evaluation metric might be expected to perform well by another metric, we saw that this was generally not the case for traditional evaluation metrics and the clinical utility score that we devised for the Challenge. **Figure 3*A*** compares each algorithm’s area under the receiver operating characteristic curve (AUROC) with its utility score on the test sets from each of the hospital systems. AUROC and utility scores are positively correlated on test sets A and B (Spearman rank correlation coefficients *ρ* = 0.791 and *ρ* = 0.839, respectively). These scores are poorly correlated on test set C (Spearman rank correlation coefficient *ρ* = 0.054), which corresponds to the hospital system for which participants did not receive training data. Furthermore, even on test sets A and B, algorithms with high utility scores did not necessarily have high AUROC scores, demonstrating that traditional evaluation metrics do not necessarily capture the clinical utility of predictions.

Furthermore, the choice of evaluation metric influenced how transferable algorithms appeared to be across hospital systems. **Figure 3**, ***B*–*D*** compares each algorithm’s AUROC or utility score on test sets from different hospital systems. Although AUROC scores are strongly correlated for each pair of hospital systems (Spearman rank correlation coefficients *ρ* = 0.973 for hospital systems A and B, *ρ* = 0.938 for hospital systems A and C, and *ρ* = 0.947 for hospital systems B and C), this is not true for utility scores. Utility scores are strongly correlated between the two hospital systems for which we provided training data (Spearman rank correlation coefficient *ρ* = 0.949 for test sets A and B), but they are poorly correlated with the third hospital system for which we did not provide training data (Spearman rank correlation coefficients *ρ* = –0.033 and *ρ* = 0.013 for hospital systems A and B, respectively, with hospital system C). **Figure 3*E*** further shows that the methods with the highest scores on data from hospital systems with shared training databases were not necessarily the methods with the highest scores on the hidden database from a separate hospital system.

Our use of clinical data from multiple hospital systems and our application of a clinical utility-based evaluation metric provided a more nuanced view of predictive generalizability than results on one system with traditional evaluation metrics would present.

## DISCUSSION

The PhysioNet/Computing in Cardiology Challenge 2019 asked participants to develop automated, open-source algorithms for the early detection of sepsis from clinical data. We assembled over 60,000 patient records from three hospital systems, with two shared publicly and one remaining hidden. By posting two databases publicly, we provided participants the opportunity to create training methodologies that do not overfit to one medical center. The third hidden database provided a strong indication of how well participants had accomplished this critical task.

We also proposed and used a novel evaluation metric that captures the clinical utility of early sepsis detection, weighted by the relative “earliness” or “lateness” of each prediction.

We suggest that this metric should be considered for wider adoption in clinical care because it does not suffer from many of the problems of *F*-measures (and related metrics such as accuracy, sensitivity, and positive predictive value) or standard AUC metrics (such as AUROC and area under the precision recall curve), which either assume a one-shot decision or no decision threshold, respectively. In particular, this novel evaluation metric shows that algorithms that perform well in one hospital system may perform poorly in another.

A third novelty in this Challenge is the development of graphical and analytical approaches to measure the similarity between entries between supposedly independent Challenge teams. We identified and disqualified teams that appeared to be highly related to each other and did not provide satisfactory explanations of these relationships.

We received 853 entries from 104 participants in academia and industry, providing a diverse view of algorithmic approaches to early sepsis detection. Combined, these efforts provide a more complete picture of how algorithms can provide early sepsis predictions. A subsequent analysis of the best performing and most interesting algorithms submitted to the Challenge will combine the strengths of different approaches to push the boundaries of automated approaches to early sepsis prediction.

## Supplementary Material
