# Supplementary material for: Early Prediction of Sepsis From Clinical Data: The PhysioNet/Computing in Cardiology Challenge 2019
Source: Crit Care Med. 2020 Jan 15;48(2):210–7. doi: 10.1097/CCM.0000000000004145 (PMC6964870; doi:10.1097/CCM.0000000000004145)
Supplement: Supplementary file 1 [file ccm-48-0210-s001.docx]

| **Hospital system** | **A** | **B** |
| --- | --- | --- |
| Number of patients | 20,336 | 20,000 |
| Number of septic patients | 1,790 | 1,142 |
| Sepsis prevalence | 8.8% | 5.7% |
| Number of rows | 739,663 | 684,508 |
| Number of entries | 5,536,849 | 4,950,064 |
| Density of entries | 20.6% | 19.1% |

Supplemental Table 1: **Data summary**. Summary of vital sign (rows 1-8 in the data set) and laboratory value (rows 9-34) data in the shared datasets for hospital systems A and B.
