# Supplementary figures and images for: Early Prediction of Sepsis From Clinical Data: The PhysioNet/Computing in Cardiology Challenge 2019
Source: Crit Care Med. 2020 Jan 15;48(2):210–7. doi: 10.1097/CCM.0000000000004145 (PMC6964870; doi:10.1097/CCM.0000000000004145)

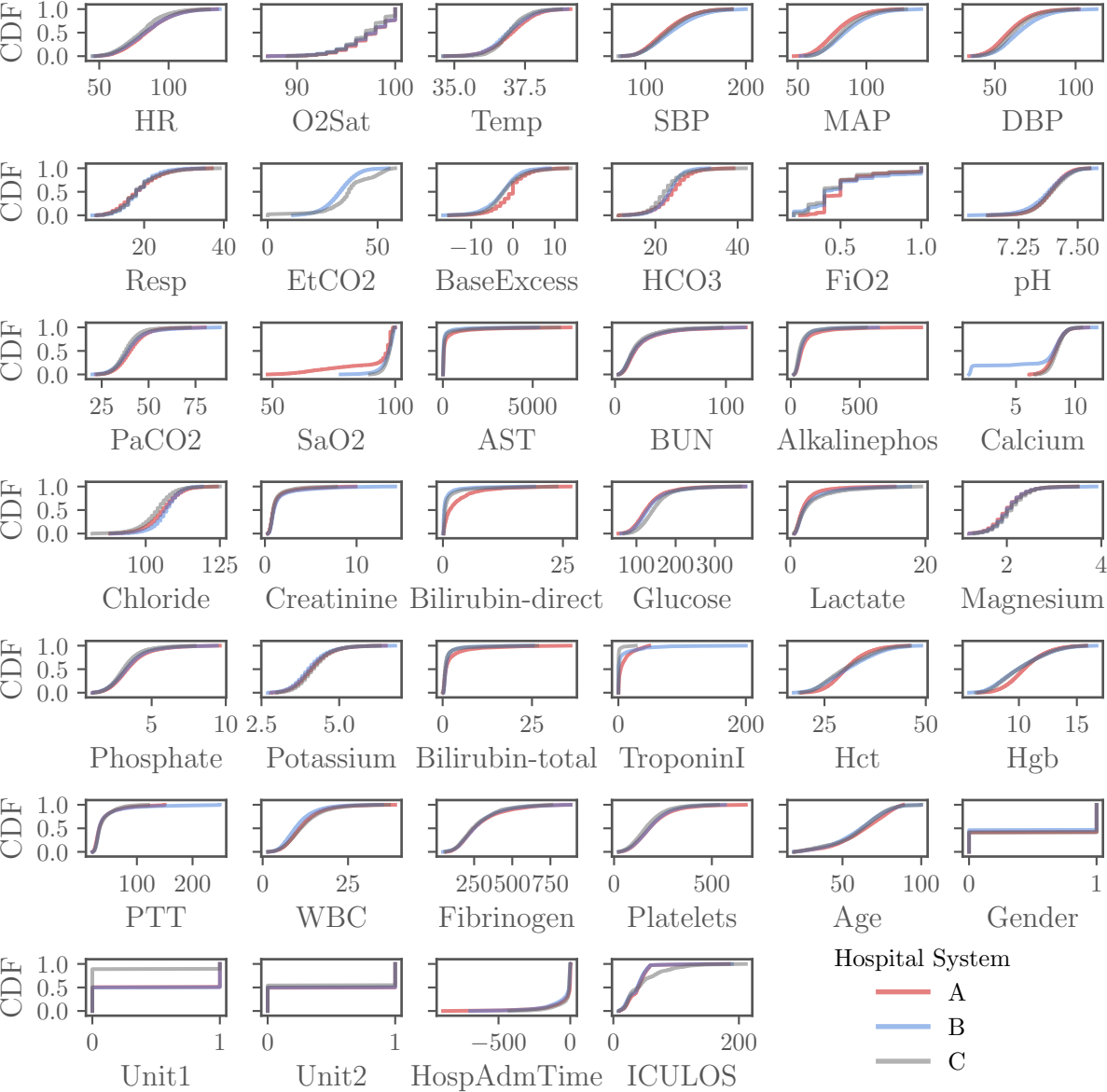

Supplement: Supplementary file 2 [file ccm-48-0210-s002.pdf]

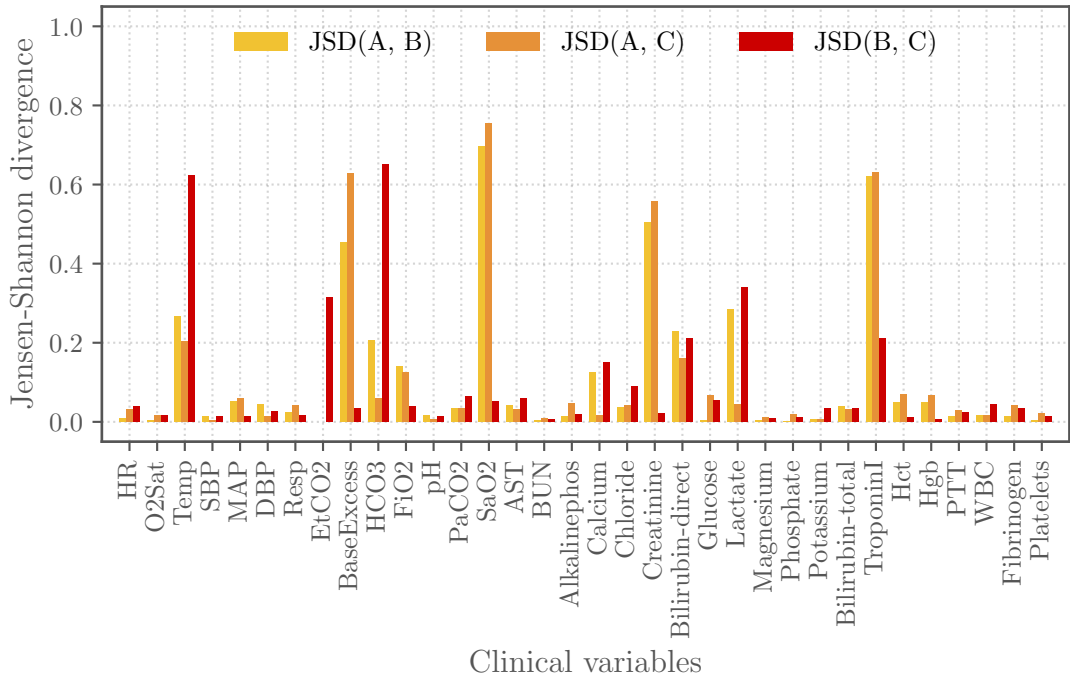

Supplement: Supplementary file 3 [file ccm-48-0210-s003.pdf]

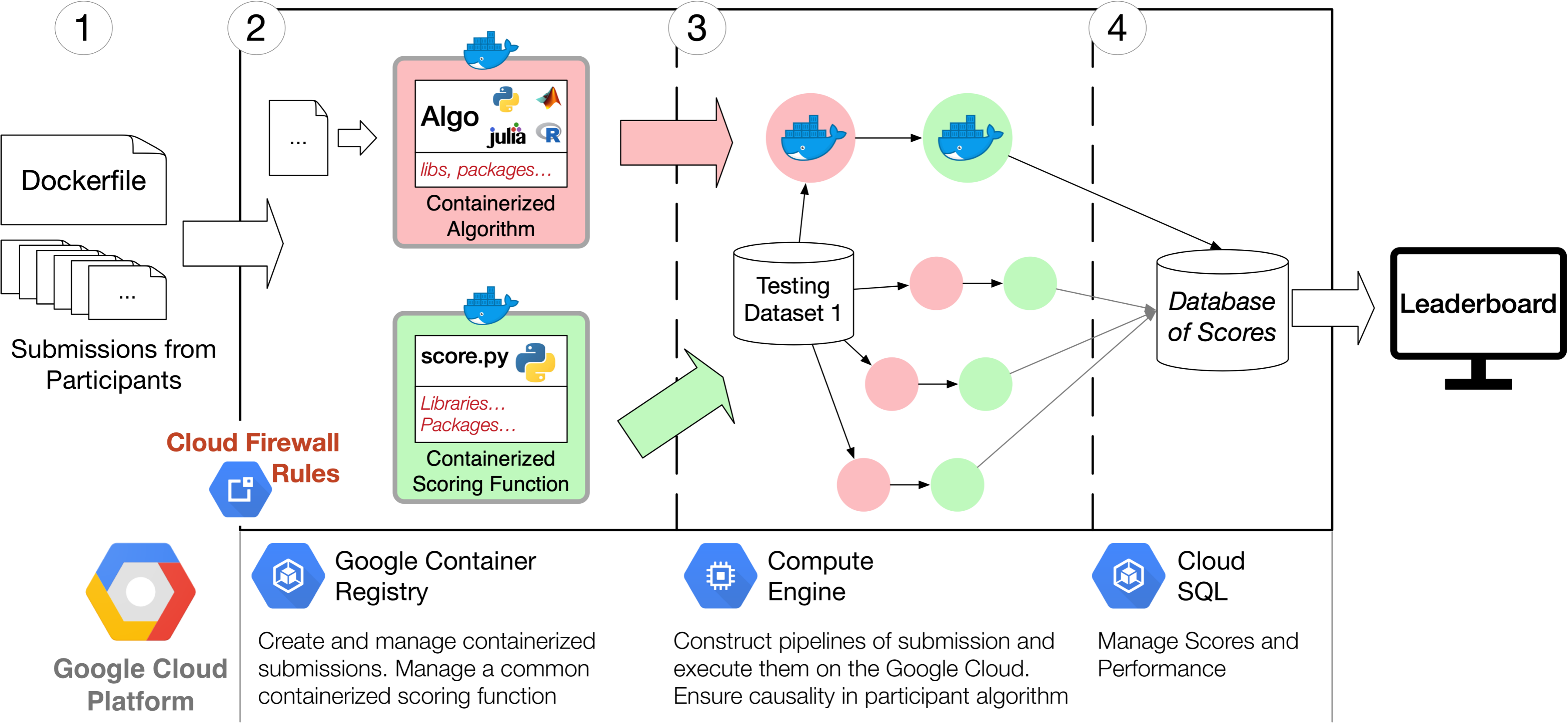

Supplement: Supplementary file 4 [file ccm-48-0210-s004.pdf]

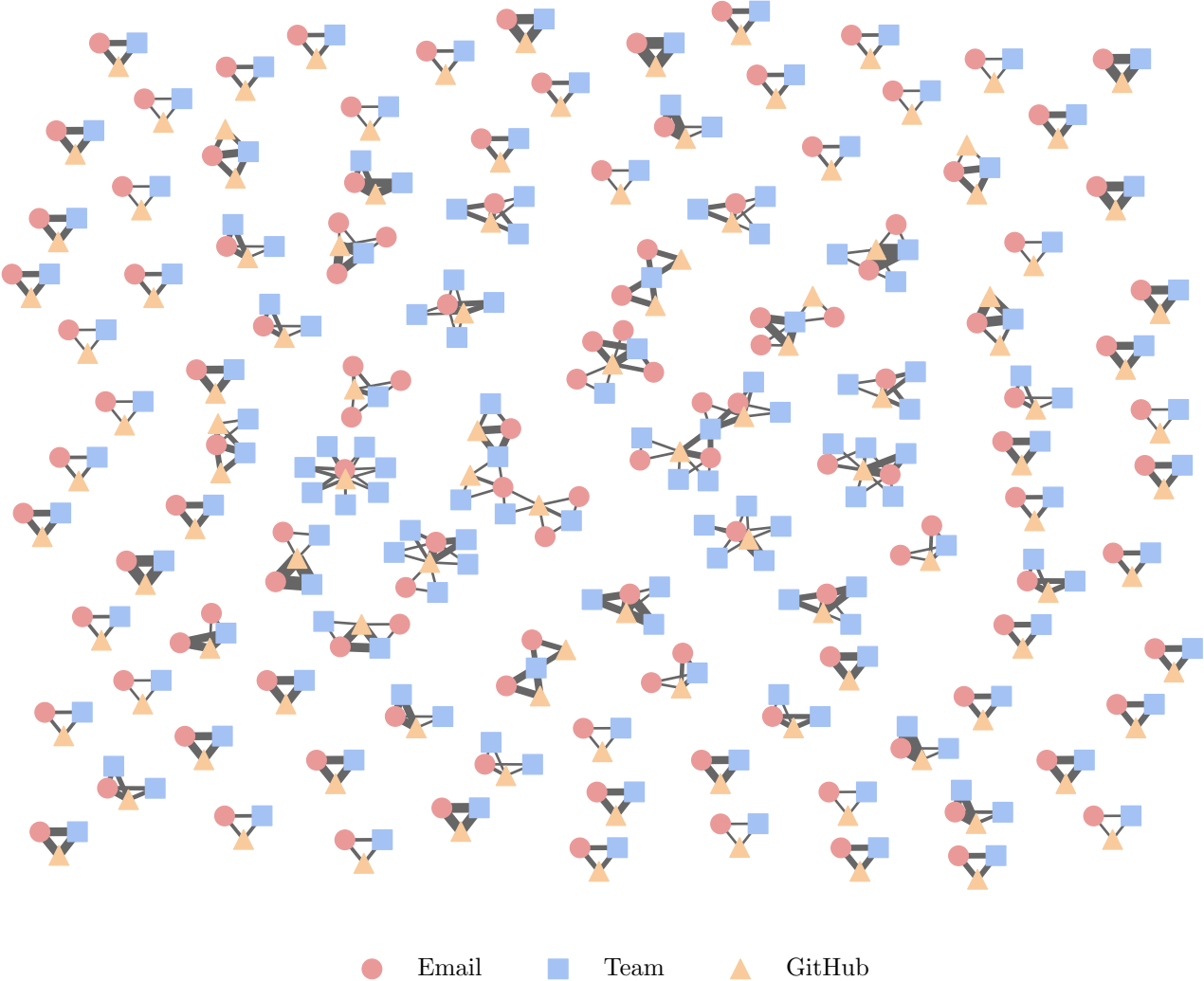

Supplement: Supplementary file 5 [file ccm-48-0210-s005.pdf]
